# Characterization of *Staphylococcus aureus* Isolated from Non-Native Patients with Skin and Soft Tissue Infections in Shanghai

**DOI:** 10.1371/journal.pone.0123557

**Published:** 2015-04-29

**Authors:** Fei-Fei Gu, Qi Hou, Hai-Hui Yang, Yue-Qiu Zhu, Xiao-Kui Guo, Yu-Xing Ni, Li-Zhong Han

**Affiliations:** 1 Department of Clinical Microbiology, Ruijin Hospital, Shanghai Jiao Tong University School of Medicine, Shanghai, China; 2 Laboratory, Shanghai United Family Hospital, Shanghai, China; 3 Department of Clinical Laboratory, Renji Hospital, Shanghai Jiao Tong University School of Medicine, Shanghai, China; 4 Department of Microbiology and Parasitology, Shanghai Jiao Tong University School of Medicine, Shanghai, China; Rockefeller University, UNITED STATES

## Abstract

**Background:**

*Staphylococcus aureus* is one predominant cause of skin and soft-tissue infections (SSTIs), but little information exists regarding the characterization of *S*. *aureus* from non-native patients with SSTIs in China.

**Methods:**

In this study, we enrolled 52 non-native patients with *S*. *aureus* SSTIs, and 65 native control patients with *S*. *aureus* SSTIs in Shanghai. 52 and 65 *S*. *aureus* isolates were collected from both groups, respectively. *S*. *aureus* isolates were characterized by antimicrobial susceptibility testing, toxin gene detection, and molecular typing with sequence type, *spa* type, *agr* group and SCC*mec* type.

**Results:**

Methicillin-resistant *S*. *aureus* (MRSA) was detected in 8 non-native patients and 14 native patients with SSTIs. Overall, antimicrobial susceptibilities of *S*. *aureus* isolated from non-native patients were found higher than those from native patients. CC59 (ST338 and ST59) was found in a total of 14 isolates (4 from non-native patients; 10 from native patients), 9 of which were carrying *lukS/F-PV* (3 from non-native patients; 6 from native patients). ST7 was found in 12 isolates and all 12 isolates were found in native patients. The livestock-associated clone ST398 was found in 11 isolates (6 from non-native patients; 5 from native patients), and 5 ST398 *lukS/F-PV*-positive methicillin-susceptible *S*. *aureus* (MSSA) were all discovered among non-native patients. The molecular epidemiology of *S*. *aureus* isolated from non-native patients was quite different from those from native patients. *lukS/F-PV* was more frequent in isolates originating from non-native patients with SSTIs compared to native patients (31 vs. 7, P <0.0001).

**Conclusions:**

CC59 was the most common clonal complex among patients with SSTIs in Shanghai. The other most common sequence types were ST7 and Livestock ST398. The molecular epidemiology of *S*. *aureus* isolated from non-native patients was quite different from those from native patients. *S*. *aureus* isolated from non-native patients was more likely to carry *lukS/F-PV*.

## Introduction

Skin and soft tissue infections(SSTIs) are common and range in severity from minor, self-limiting, and superficial infections to life-threatening diseases requiring all resources of modern medicine[1].*Staphylococcus aureus* currently is the leading cause of SSTIs across all continents[2, 3].MRSA was first discovered in the 1960s, and quickly became a critical pathogen in hospitals globally, leading to the emergence of healthcare-associated MRSA (HA-MRSA). Community-associated MRSA (CA-MRSA) subsequently appeared in Chicago, among other part of North America, in the late 1980s and early 1990s[4].Infection of the skin and soft tissues is by far the most frequent disease manifestation associated with CA-MRSA, accounting for at least 90% of all CA-MRSA infections[5].

*S*. *aureus* contains a wide variety of enzymes and toxins including the Panton-Valentine leukocidin(PVL). PVL is one toxin produced by *S*. *aureus*, and consists of two components with subunits LukS-PV and LukF-PV which are secreted individually by the bacteria before forming a heptamer that induces pores in polymorphonuclear leukocytes [6].PVL may well be the key toxin in SSTIs, but there is some debate about whether it is the key pathogenic factor in necrotic SSTIs and necrotic haemorrhagic pneumonia, or remains just as a marker of such disease-causing potential. Other virulence factors playing a role in SSTIs include coagulase, protein A, toxic shock syndrome toxin (TSST1) and scalded skin syndrome toxin (SSST)[7].

In China, ST59-MRSAIV/V-t437 was the most common clone among CA-MRSA isolates reported among children with SSTIs[8, 9].One recent study in Beijing reported the livestock ST398 clone as having a high prevalence in *S*. *aureus* SSTIs, where 64.3% of ST398 strains were harboring the *lukS/F-PV* gene[10]. In Japan, the most common clone among SSTI-associated MRSA was MLST-CC8/spa-CC008-SCC*mec*-IV [11].CC8 is the most common type in *S*. *aureus* SSTIs, especially among PVL-positive *S*. *aureus* as reported in New York, the United States[12].

Shanghai is a large, dynamic city in China where about 200,000 foreigners reside, many of whom lead different lifestyles and receive much different medical care in comparison to the native Chinese population. To our knowledge, there exists no information characterizing the differences, if any, of *S*. *aureus* in SSTIs between non-native patients and native patients which respect to antimicrobial resistance, toxin production and gene typing. Our study aims to discover any significant differences in antimicrobial susceptibility, toxin gene detection and molecular typing of *S*. *aureus* between non-native and native patients with SSTIs.

## Materials and Methods

### Patients and Bacterial isolates

A total of 117 patients, including 52 non-native patients and 65 native patients, from three hospitals (Hospital A, B and C) with SSTIs were enrolled in this study. All 117 *S*. *aureus* isolates were collected from these patients during the period of January 2011 to November 2013. Hospital A is a joint venture hospital mainly serving foreigners in Shanghai, while Hospital B and C are tertiary public hospitals in Shanghai that mainly serve native patients. Native patients in our study were defined as patients who were born in Mainland China, live permanently in Shanghai and carry publicly provided medical insurance. Non-native patients were defined as those who originated from other countries or regions, and short-term or long-term stay in Shanghai for employment or travel. Non-native patients do not carry publicly provided medical insurance, and instead generally seek medical care from specific hospitals (like Hospital A in our study) that accept health plans offered by private international insurance carriers. Four patients from Hong Kong SAR of PRC in Hospital A in this study conform to the definition of non-native patients and carried private international insurance. They were enrolled in the non-native group.

Fifty-two *S*. *aureus* isolates from 52 non-native patients with SSTIs in Hospital A were collected. Sixty-five *S*. *aureus* isolates collected from 65 native patients with SSTIs were from Hospitals B and C, and served the controls in this study. The SSTIs we studied were considered superficial, uncomplicated infections, including impetigo, erysipelas and cellulitis. All 117 *S*. *aureus* isolates enrolled in this study were identified by a combination of phenotypic tests as previously described[13].

CA-MRSA is defined as MRSA isolated from an outpatient or within 2 days of a patient’s hospitalization. Exclusion criteria included a history of hospitalization for illness, surgery, or dialysis in the previous year or the presence of indwelling catheters or other medical devices. HA-MRSA was defined by isolation >2 days after hospitalization or presence of any of the aforementioned healthcare risks[14].

This study was approved by the Ruijin Hospital Ethics Committee (Shanghai Jiao Tong University School of Medicine).The Review Board exempted the need for informed consent for this retrospective study.

### Antimicrobial susceptibility testing

Antimicrobial susceptibility profiles of *S*. *aureus* isolates were determined by the disk diffusion method, according to the guidelines of Clinical and Laboratory Standards Institute (CLSI)[15]. Antibiotics tested included penicillin (10 units), cefoxitin (30 μg), gentamicin (10 μg), kanamycin (30μg), tobramycin (10 μg), erythromycin (15 μg), tetracycline (30μg), teicoplanin (30μg), minocycline (30μg), ciprofloxacin (5μg), clindamycin (2μg), sulfamethoxazole-trimethoprim (25 μg), chloramphenicol (30μg), rifampicin (5μg), quinupristin-dalfopristin (15μg), linezolid (30 μg), fusidic acid (10 μg) and mupirocin (5μg and 200μg).The minimum inhibitory concentration (MIC) of vancomycin was detected by E-test. The penicillin disk diffusion zone edge test was performed for β-lactamase detection, and inducible clindamycin resistance was determined by the D-test. *S*. *aureus* ATCC25923 and ATCC29213 were used as quality controls for the disk diffusion test and MIC detection respectively.

### Detection of toxin genes

A variety of clinically significant toxin genes were detected by PCR, including *lukS/F-PV* (encoding Panton-Valentine leukocidin); *tst* (encoding toxic shock syndrome toxin 1); *eta* and *etb* (encoding exfoliative toxin A and B); *sea*-*see* and *seg*-*sej* (encoding staphylococcal enterotoxins SEA-SEE and SEG-SEJ)[16]; and *sasX* (encoded in a mobile genetic element) which also acts as a virulence determinant and plays a key role in MRSA colonization and pathogenesis as recently reported by Li *et al*.[17].

### Detection of molecular types

DNA was extracted by the simplified alkaline-lysis method[13]. All *S*. *aureus* isolates were investigated by multilocus sequence typing (MLST), *spa* typing and accessory gene regulator (*agr*) typing as previously described [18]. MRSA isolates were confirmed by the *mec*A gene, and SCC*mec* types of MRSA were determined by a method previously described [19].

### Statistical analysis

The chi-square or Fisher’s exact test were used where appropriate using the software package SAS 8.2 (SAS Institute Inc., Cary, NC, USA). A two-sided P value of < 0.05 was considered statistically significant.

## Results

### Clinical data

The median age of non-native patients and native patients was 30 years (range: 2–56 years) and 48 years (range: 8–84 years) respectively, while the sex distribution (male/ female) was 32/20 (61.5%/38.5%) and 35/30 (53.8%/46.2%) respectively. Among the non-native patients, 51.9% were outpatients, whereas as 43.1% of the native patients were considered outpatients. Abscesses were the most common infection type in both groups.

The nationalities of origin of non-native patients include the United States (11/52, 21.2%), Germany (10/52, 19.2%), France (6/52, 11.5%),Hong Kong (4/52, 7.7%), the United Kingdom (3/52, 5.8%), Australia (3/52, 5.8%),Austria (2/52, 3.8%),Belgium (2/52, 3.8%), Japan (2/52, 3.8%),India (2/52, 3.8%),Brazil (1/52, 1.9%),Italy (1/52, 1.9%), Spain (1/52, 1.9%), Finland (1/52, 1.9%),the Netherlands (1/52, 1.9%),Norway (1/52, 1.9%) and Sweden (1/52, 1.9%).

### Antimicrobial susceptibility testing

Ninety-five isolates (44 from non-native patients; 51 from native patients) were MSSA, and 22 isolates (8 from non-native patients; 14 from native patients) were confirmed as MRSA. All 117 isolates were susceptible to quinupristin-dalfopristin, linezolid and vancomycin. Only one MRSA isolated from a native patient showed intermediate susceptibility to teicoplanin. Sixteen isolates (13.7%) were susceptible to penicillin with β-lactamase negative. Seven isolates (4 MRSA and 3 MSSA from native patients) were observed showing high-level mupirocin resistance and 5 (4 MRSA and 1 MSSA) of which all belong to ST764. A total of 25 isolates (1 MRSA and 9 MSSA from non-native patients, 4 MRSA and 11 MSSA from native patients) were inducibly resistant to clindamycin based on D-test results. Antimicrobial susceptibilities of *S*. *aureus* isolated from non-native patients were mostly higher than those from native patients. These findings are summarized in Table 1.

**Table 1:** Antimicrobial susceptibilities of *S*. *aureus* isolated from non-native patients and native patients with skin and soft tissue infections (SSTIs).

Antimicrobial	Non-native patients (%)		Native patients (%)	
	MRSA	MSSA	MRSA	MSSA
Penicillin	0	25.0	0	9.8
Oxacillin	0	100	0	100
Gentamicin	87.5	100	57.1	80.4
Kanamycin	37.5	88.6	0	60.8
Tobramycin	87.5	95.5	50.0	66.7
Erythromycin	37.5	72.7	0	54.9
Tetracycline	87.5	93.2	14.3	84.3
Teicoplanin	100	100	92.9	100
Minocycline	100	100	50.0	100
Ciprofloxacin	75.0	97.7	28.6	88.2
Clindamycin^a^	75.0	95.5	7.1	74.5
Sulfamethoxazole-trimethoprim	100	100	92.9	100
Chloramphenicol	100	93.2	64.3	80.4
Rifampicin	100	100	85.7	100
Quinupristin-dalfopristin	100	100	100	100
Linezolid	100	100	100	100
Fusidic acid	100	97.7	92.9	96.1
Mupirocin^b^	100	100	71.4	94.1
Vancomycin	100	100	100	100

^a^25 isolates (1 MRSA and 9 MSSA from non-native patients, 4 MRSA and 11 MSSA from native patients respectively) were D-test positive, indicating inducible clindamycin resistance.

^b^7 isolates (4 MRSA and 3 MSSA from native patients) presented high-level mupirocin resistance.

### Virulence factors

As presented in Table 2, *seg* and *sei* were the most frequently found of the enterotoxin genes that we detected, occurring in 42 and 43 isolates respectively. *lukS/F-PV* was found in 38 isolates, of which 31 isolates (8 MRSA and 23 MSSA) were from non-native patients and7 isolates (4 MRSA and 3 MSSA) were from native patients. No *see*-positive isolates were detected in non-native patients or native patients. *S*. *aureus* isolates from non-native patients were more likely to carry *lukS/F-PV*, *seg* and *sei* (P<0.0001, P = 0.0045 and P = 0.0023 respectively), while those from native patients tended to carry *sea* (P = 0.0409).

**Table 2:** Prevalence of virulence genes from 117 *S*. *aureus* isolates from non-native patients and native patients with skin and soft tissue infections (SSTIs).

	Total (n = 117) n(%)	Non-native patients (n = 52) n (%)	Native patients (n = 65) n (%)	P-value
*lukS/F-PV*	38 (32.5)	31 (59.6)	7(10.8)	<0.0001
*tst*	10 (8.5)	4 (7.7)	6 (9.2)	1.0000
*eta*	5 (4.3)	2 (3.8)	3 (4.6)	1.0000
*etb*	4 (3.4)	2 (3.8)	2 (3.1)	1.0000
*sea*	12 (10.3)	2 (3.8)	10 (15.4)	0.0409
*seb*	17 (14.5)	6 (11.5)	11 (16.9)	0.4115
*sec*	14 (12.0)	4 (7.7)	10 (15.4)	0.2027
*sed*	4 (3.4)	2 (3.8)	2 (3.1)	1.0000
*see*	0	0	0	-
*seg*	42 (34.2)	26 (50.0)	16 (24.6)	0.0045
*seh*	2 (1.7)	0	2 (3.1)	0.4993
*sei*	43 (36.8)	27 (51.9)	16 (24.6)	0.0023
*sej*	4 (3.4)	2 (3.8)	2 (3.1)	1.0000
*sasX*	2 (1.7)	0	2 (3.1)	0.4993

*lukS/F-PV*, gene encoding Panton-Valentine leukocidin; *tst*, gene encoding toxic shock syndrome toxin 1; *eta* and *etb*, gene encoding exfoliative toxin A and B; *sea*-*see* and *seg*-*sej*, gene encoding staphylococcal enterotoxins SEA-SEE and SEG-SEJ; *sasX*, gene encoding mobile genetic element; P-value, two-sided P-value calculated by the chi-square or Fisher’s exact test as appropriate.

### Molecular epidemiologic characteristics

**Fig 1:**
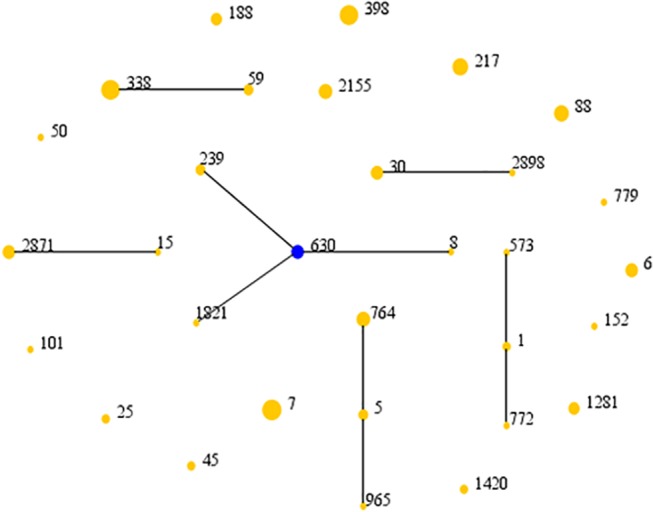
The rough sketch produced by eBURST with the stringent (default) group definition, representing the *S*. *aureus*population and the positions of 117 isolates. These isolates were classified into six groups and fifteen singletons. Each number represents an MLST ST. The ST of the predicted founder is represented by a blue circle and the area of each circle indicates the prevalence of the ST in the MLST data of this study.

Among the 117 *S*. *aureus* isolates, 31 sequence types (STs) and 59 *spa* types were identified (S1 Table). We detected 8 SCC*mec* type I, 4 SCC*mec* type II, 2 SCC*mec* type III, 3 SCC*mec* type IV, and 4 SCC*mec* type V isolates. One MRSA isolate could not be SCC*mec* typed. As seen in Table 3, MRSA and MSSA isolates from both non-native patients and native patients expressed a great diversity in molecular epidemiologic characteristics. Only one specific clone, ST338-SCC*mec*V-t437 was present in MRSA isolates from both non-native patients and native patients. As shown in Table 3, we discovered a few characteristics to be similar among clones from *S*. *aureus* isolates from non-native patients and native patients. The most common ST was ST7 (12/117, 10.3%), followed by ST338 (11/117, 9.4%), ST398 (11/117, 9.4%) and ST217 (8/117, 6.8%). All 12 ST7 isolates were sourced from native patients, and all 8 ST217 isolates were isolated from non-native patients. ST338 was discovered in 3 isolates from non-native patients and 8 isolates from native patients, and ST398 was found in 6 isolates from non-native patients and 5 isolates from native patients.ST338 was the most common ST in *lukS/F-PV*-positive isolates, including 3 isolates (2 MRSA and 1 MSSA) from non-native patient and 5 isolates (4 MRSA and 1 MSSA) from native patients. The other most common STs in *lukS/F-PV*-positive isolates were ST217 (6 MSSA), ST398 (5 MSSA) and ST88 (4 MSSA and 1 MRSA), and these 16 isolates all originated from non-native patients. The relationships of 117 *S*. *aureus* isolates are shown in a diagram produced by eBURST based on the MLST data of this study, and are shown in Fig 1. These isolates were classified into six groups and fifteen singletons with the stringent (default) group definition of six or more shared alleles. The main groups contain CC8 (ST8, ST239, ST630 and ST1821), CC59 (ST59 and ST338) and CC5 (ST5, ST764 and ST965).

**Table 3:** Distribution of clones among 117 *S*. *aureus* isolates from non-native patients and native patients with skin and soft tissue infections (SSTIs).

Clone	CCs	*agr* group	Total (n = 117)	Non-native patients (n = 52)	Native patients (n = 65)	Virulence genes (n)
ST239-SCC*mec*III-t030/ t037	8	I	2	0	2	*sea* (2), *sasX*(1)
ST338-SCC*mec*I-t437	59	I	4	0	4	*lukS/F-PV* (2), *sea*(1),*seb*(3)
ST5-SCC*mec*II-t2460	5	II	1	0	1	*tst* (1), *sec* (1), *seg* (1),*sei* (1)
ST764-SCC*mec*II-t002	5	II	3	0	3	*tst* (3), *sec* (3), *seg*(3), *sei* (3)
ST764-SCC*mec*NT-t002	5	II	1	0	1	*seb* (1), *seg* (1), *sei* (1)
ST965-SCC*mec*I-t062	5	II	1	0	1	*sea* (1), *seg* (1), *sei* (1)
ST338-SCC*mec*V-t437	59	I	3	1	2	*lukS/F-PV* (3), *seb*(3)
ST338-SCC*mec*IV-t437	59	I	1	1	0	*lukS/F-PV* (1)
ST772-SCC*mec*V-t4599	1	II	1	1	0	*lukS/F-PV* (1),*sea* (1), *sec* (1), *seg* (1), *sei* (1)
ST30-SCC*mec*I-t019	30	III	1	1	0	*lukS/F-PV*(1), *seg* (1), *sei* (1)
ST30-SCC*mec*IV-t019	30	III	2	2	0	*lukS/F-PV* (2), *seg* (2), *sei* (2)
ST88-SCC*mec*I-t1814	88	I	1	1	0	*lukS/F-PV* (1)
ST8-SCC*mec*I-t5160	8	I	1	1	0	*lukS/F-PV* (1)
ST15-t853	15	II	1	0	1	None
ST1821-t13742	8	I	1	0	1	None
ST188-t189/ t2883	188	I	4	0	4	*tst* (1), *seb* (1), *sec* (2)
ST1-t127	1	III	2	0	2	*sea* (1),*sec* (2), *seh* (2)
ST2155-t13740	121	IV	1	0	1	*eta* (1), *etb*(1), *seg* (1), *sei*(1)
ST239-t074	8	I	1	0	1	*sasX* (1)
ST30-t318	30	III	1	0	1	*lukS/F-PV* (1), *seg* (1), *sei* (1)
ST398-t1451/ t7880	398	I	3	0	3	None
ST59-t172	59	I	1	0	1	*lukS/F-PV* (1), *sea* (1)
ST5-t045	5	II	1	0	1	*seg* (1), *sei* (1)
ST573-t1839	1	II	1	0	1	*seg* (1), *sei* (1)
ST630-t377	8	I	4	0	4	None
ST764-t002	5	II	2	0	2	*sec*(1), *sed*(2), *seg* (2),*sei* (2), *sej*(2)
ST7-t091/ t13741/ t3932/ t796	7	I	12	0	12	*tst*(1), *etb* (1)
ST88-t13739/ t2592	88	III	2	0	2	None
ST1281-t164	20	I	4	2	2	*seg* (4), *sei* (4)
ST25-t078	25	I	2	1	1	*seb* (2), *sec* (1^no^), *seg* (2), *sei* (2)
ST2871-t084	15	II	5	4	1	*eta* (1^no^)
ST338-t437	59	I	3	1	2	*lukS/F-PV* (2^a^), *seb* (2^n^)
ST398-t034/ t571	398	I	6	4	2	*lukS/F-PV* (4^no^), *eta* (1^n^)
ST45-t8232	45	I	2	1	1	*sec* (2), *seg* (2), *sei* (2)
ST59-t163	59	I	2	1	1	*sea* (1^no^),*seb* (2)
ST6-t701	6	I	5	1	4	*sea* (4^n^), *eta* (1^n^)
ST101-t056	101	I	1	1	0	None
ST1420-t2835	Singleton	II	2	2	0	*lukS/F-PV*(2), *seg* (2), *sei*(2)
ST152-t355	152	I	1	1	0	*lukS/F-PV* (1), *sed*(1), *sej* (1)
ST2155-t1425/ t159/ t308/ t645	121	IV	5	5	0	*lukS/F-PV* (3), *eta* (1), *etb* (2), *seb* (2), *seg* (5), *sei*(5)
ST217-t005/ t2986/ t309/ t9181	22	I	8	8	0	*lukS/F-PV* (6), *tst*(2), *sec* (1), *seg* (8), *sei* (8)
ST2898-t021	30	III	1	1	0	*tst* (1),*seg*(1), *sei*(1)
ST30-t012	30	III	1	1	0	*tst* (1), *seg* (1), *sei* (1)
ST398-t011/ t6605	398	I	2	2	0	*lukS/F-PV* (1)
ST50-t185	50	IV	1	1	0	*sei*(1)
ST5-t105	5	II	1	1	0	*lukS/F-PV* (1),*seg* (1),*sei* (1)
ST630-t12148	8	I	1	1	0	None
ST779-t878	779	III	1	1	0	*seb* (1), *sed* (1), *sej* (1)
ST88-t1376/ t6497	88	III	4	4	0	*lukS/F-PV* (4)

^no^ the isolate(s) was (were) found in non-native patients.

^n^ the isolate(s) was (were) found in native patients.

^a^1*lukS/F-PV*-positive isolate was found in non-native patients and the other was found in native patients.

Clone, ST-SCC*mec*-*spa*-type; ST, sequence type by multi-locus sequence typing; SCC*mec*, Staphylococcal cassette chromosome *mec*; *spa*, Staphylococcus protein A gene; NT, not-typeable; CCs, clonal complexes;*agr*, accessory gene regulator; None, no virulence gene detected.

*agrI* was the most frequent *agr* group found in isolates from both non-native patients and native patients (27/52, 51.9%; 47/65, 72.3%respectively), but was found more prevalent in native patients (P = 0.0231). In contrast, *agrIII* and *agrIV* were found more likely to occur in non-native patients (11 vs. 5, P = 0.0352; 6 vs. 1, P = 0.0234, respectively). *agrII* was detected in 8isolates from non-native patients and 12 isolates from native patients (P = 0.6604).

## Discussion

*S*. *aureus* particularly CA-MRSA is the predominant cause of SSTIs worldwide[20].In our study, 22 isolates (18.8%) of 117 isolates were found as MRSA, and it was lower than the occurrences of MRSA among *S*. *aureus* SSTIs previously reported[1, 2, 21].Eight MRSA isolates (3 HA-MRSA and 5 CA-MRSA) were found among non-native patients and 14 MRSA isolates (12 HA-MRSA and 2 CA-MRSA) were found among native patients. We found no significant difference between the occurrences of MRSA in non-native patients compared to native patients. Nevertheless, as shown in Table 1, the isolates from non-native patients showed higher antimicrobial susceptibilities than those from native patients, and high-level mupirocin resistance was detected only in 7 isolates from native patients. High-level mupirocin resistance is mediated by the *mupA* gene, which is located on plasmids that vary in size, restriction patterns and ability to transfer by conjugation[22]. In this study, *mupA* was detected in the 7 high-level mupirocin resistance isolates and found positive in 5 isolates. Of 5 *mupA*-positive isolates, 4 isolates were ST764 (3 MRSA and 1 MSSA) and one isolate was ST338 MSSA. These resistance findings among native patients are probably due to overuse of antibiotic therapy which commonly occurs in public hospitals in China. The different occurrences of HA- and CA-MRSA between non-native and native patients may also imply the different routes of MRSA infection among these two groups. It is likely that non-native patients are more likely to contract CA-MRSA SSTIs in comparison to native patients according to the feature of MRSA as previously described, whereas native patients are more frequently infected with HA-MRSA SSTIs. As common clone lineages in MRSA, ST338-t437was usually associated to SCC*mec* types IV or V, and ST30-t019 was usually associated to SCC*mec*IV. However, in this study we have found 4 ST338-t437 MRSA isolates and 1 ST30-t019MRSA isolate that associated to SCC*mec*I.

Ever since its emergence in 2000 in the United States, prevalence ofST8 (USA300) has led to a high burden of SSTIs globally[23], as witnessed by *S*. *aureus* clone outbreaks among SSTIs in Japan, Korea, Singapore and Europe[11, 24–27].We found one ST8 isolate from a non-native patient in our study as ST8-SCC*mec*I-t5160 with *lukS/F-PV* positive. However, CC8 (ST8, ST239, ST630 and ST1821) was found in 10 isolates, of which 2 (1 MRSA and 1MSSA) were from non-native patients and 8 (2 MRSA and 6 MSSA) were from native patients. CC8 might also be an important clonal complex among SSTIs in China especially in native patients. The occurrence of CC8 was significantly higher among native patients in comparison to non-native patients (8vs. 2, P = 0.0230). ST239 is recognized as a common epidemic clone in bloodstream infections in China[18] and ST630 was also recently reported to cause severe infective endocarditis with systemic embolism in China[28].ST7,found in a total of 12 MSSA isolates, was the most common ST in our study. ST7 has also been found to be among the most common genotypes of MSSA in invasive community-acquired *S*. *aureus* infection in Chinese children, along with ST88, ST25, ST2155 and ST188[29]. As shown in Table 3, the clone characteristics of *S*. *aureus* isolated from non-native patients and native patients were quite different. The molecular characterization of isolates from non-native patients did not show any centralized tendency or distinguishing features related to the countries (regions) or continents of patients’ origin.

A study by Zhao *et al*. from Beijing reported that the livestock-associated ST398clone in MSSA was the most prevalent PVL-positive clone in SSTIs [10]. Similarly, ST398 in our study was found in 11 MSSA isolates (6 from non-native patients; 5 from native patients), and 5 *lukS/F-PV*-positive isolates were all from non-native patients.ST398 presented decreased human-to-human transmissibility, and we lack any clinical data on any possible patient contact with animals. Zhao *et al*. also did not find any association between livestock contact and ST398 infection[10]. However, careful monitoring of human-to-human transmissibility of ST398 of course remains important[30]. Even though there is no statistically significant difference between the ST398 isolates from non-native patients and those from native patients in our study, it is notable that the 5 *lukS/F-PV*-positive ST398 isolates were only found in non-native patients. We believe the occurrence of *lukS/F-PV* only in non-native patients is not coincidental when compared to the complete lack of *lukS/F-PV* in native patients (5/6 vs. 0/5, P = 0.0152).

PVL production by *S*. *aureus* is associated with SSTIs, most notably in a recent outbreak of PVL-positive MSSA SSTIs in France[31]. A total of 38 *lukS/F-PV*-positive isolates were discovered in our study, of which 31 isolates (8 MRSA and 23 MSSA) were from non-native patients and7 isolates (4 MRSA and 3 MSSA) were from native patients. PVL-positive *S*. *aureus* isolates were more likely to spread among non-native patients, possibly due to the different lifestyles and medical care between non-native patient and native patients. Further studies are recommended to further explore exactly why non-native patients are more likely to carry *lukS/F-PV* in comparison to native patients.

Different PVL-positive MRSA clones predominate indifferent regions, e.g., ST8 in the United States, ST30 and ST80 in Europe, and ST59 in Asia[14, 32]. As a single locus variant (SLV) of ST59, ST338 (8/38, 21.1%)was the most common *lukS/F-PV*-positive ST in our study. CC59 (ST338 and ST59) was found in a total of 14 isolates (4 from non-native patients; 10 from native patients, P = 0.0570). Nine of these 14 isolates (3 from non-native patients; 6 from native patients, P = 0.3469) carried *lukS/F-PV*. Even though ST59 was found in only 3 isolates in this study, CC59 remains the most common clonal complex among the patients with SSTIs in China when ST59 is accounted for together with ST338 (14/117, 12.0%). The other most common *lukS/F-PV*-positive STs were ST217 (6 isolates), ST398 (5 isolates) and ST88 (5 isolates), all of which were isolated from non-native patients.

## Supporting Information

S1 Table Clinical data and molecular characteristics of 117 *S*. *aureus* isolates from non-native patients and native patients with skin and soft tissue infections (SSTIs). (XLSX)
